# Single-Cell Immunogenomic Approach Identified SARS-CoV-2 Protective Immune Signatures in Asymptomatic Direct Contacts of COVID-19 Cases

**DOI:** 10.3389/fimmu.2021.733539

**Published:** 2021-11-25

**Authors:** Kaushik Sen, Sudeshna Datta, Arup Ghosh, Atimukta Jha, Abdul Ahad, Sanchari Chatterjee, Sandhya Suranjika, Soumya Sengupta, Gargee Bhattacharya, Omprakash Shriwas, Kiran Avula, Jayasingh Kshatri, Punit Prasad, Rajeeb Swain, Ajay K. Parida, Sunil K. Raghav

**Affiliations:** ^1^ Department of Infectious Disease Biology, Institute of Life Sciences (ILS), Bhubaneswar, India; ^2^ Regional Centre for Biotechnology (RCB), Faridabad, India; ^3^ School of Biotechnology, Kalinga Institute of Industrial Technology (KIIT), Bhubaneswar, India; ^4^ Manipal Academy of Higher Education, Manipal, India; ^5^ Regional Medical Research Centre (RMRC), Bhubaneswar, India

**Keywords:** SARS-CoV-2, antibody titer, scRNA-seq, scBCR-seq, scTCR-seq, cytokine levels

## Abstract

The response to severe acute respiratory syndrome coronavirus 2 (SARS*-*CoV*-*2) is largely impacted by the level of virus exposure and status of the host immunity. The nature of protection shown by direct asymptomatic contacts of coronavirus disease 2019 (COVID-19)-positive patients is quite intriguing. In this study, we have characterized the antibody titer, SARS-CoV-2 surrogate virus neutralization, cytokine levels, single-cell T-cell receptor (TCR), and B-cell receptor (BCR) profiling in asymptomatic direct contacts, infected cases, and controls. We observed significant increase in antibodies with neutralizing amplitude in asymptomatic contacts along with cytokines such as Eotaxin, granulocyte-colony stimulating factor (G-CSF), interleukin 7 (IL-7), migration inhibitory factor (MIF), and macrophage inflammatory protein-1α (MIP-1α). Upon single-cell RNA (scRNA) sequencing, we explored the dynamics of the adaptive immune response in few representative asymptomatic close contacts and COVID-19-infected patients. We reported direct asymptomatic contacts to have decreased CD4^+^ naive T cells with concomitant increase in CD4^+^ memory and CD8^+^ Temra cells along with expanded clonotypes compared to infected patients. Noticeable proportions of class switched memory B cells were also observed in them. Overall, these findings gave an insight into the nature of protection in asymptomatic contacts.

## Introduction

Since the emergence of the first coronavirus disease (COVID-19) in late December 2019, the virus has become a serious threat to mankind. It belongs to the family Coronaviridae and has ~79% sequence similarity to its counterpart, severe acute respiratory syndrome coronavirus (SARS-CoV) (1). The penetration and transmissibility of the virus *via* human-to-human contact has led to more than 248,467,363 confirmed cases of COVID-19, including 5,027,183 deaths as of 5th November, 2021, World Health Organization (WHO). WHO first declared this outbreak as a public health emergency and subsequently a global pandemic (2, 3). The infection is primarily characterized by fever, cough, fatigue, loss of taste, and smell and might range from moderate to severe acute respiratory distress syndrome (ARDS) leading to reduction in the number of proliferating lymphocytes (lymphopenia) in severe patients (4). In these patients, studies have also associated the disease with immune hyper-responsiveness called cytokine storm, characterized by increased interleukins (IL-2, IL-7, and IL-10), granulocyte-colony stimulating factor (GCSF), interferon-gamma inducible protein 10 (IP10), monocyte chemoattractant protein 1 (MCP1), and tumor necrosis factor-alpha (TNF-alpha) (4, 5). However, not all individuals exposed to SARS-CoV-2 show COVID-19 disease symptoms; few might be asymptomatic, suggesting that natural immunity can effectively combat this virus.

Understanding humoral and adaptive immunity against SARS-CoV-2 is important for vaccine development, interpretation of the disease pathogenesis, and calibration of pandemic control measures (6). Most of the studies have focused on the adaptive immune responses in COVID-19-positive patients, and as in the case of all viral infections, the role of B and T cells have been widely explored in patients (7–11). Studies on humoral immune response have shown the presence of elevated levels of IgG and IgM antibody titers in patients, the former being significantly elevated in severe patients (7). Several reports have appreciated the role of serum IgA for early neutralizing response against SARS-CoV-2 and their longevity for months after onset of symptoms (12–15). The role of T-cell immunity to SARS-CoV-2 has also been explored by researchers, and one such study elaborates on specific CD4^+^ and CD8^+^ memory T-cell responses in convalescent patients (16). Phenotyping based on *CD45RA* and *CCR7* revealed that SARS-CoV-2-specific CD4^+^ T cells were biased towards T central memory (Tcm) phenotype, whereas SARS-CoV-2-specific CD8^+^ T cells were biased toward terminally differentiated effector (Temra) cells (17). CD8^+^ Temra cells are terminally differentiated effector memory cells with low expression of *IL-2* and high expression of *IFN-γ*, showing high cytotoxicity, low proliferative capacity, and high sensitivity to apoptosis (18). In patients, these cells show high expression of T-cell activation markers and are distinct to SARS-CoV-2 virus (19).

T-cell-mediated response is also critical in mediating long-term protection against SARS-CoV-2 (20), while B-cell-mediated antibody response tends to decline in convalescent patients (21). Studies have also reported the detection of spike reactive CD4^+^ T cells not only in patients but also in unexposed individuals (22). The presence of these spike-reactive CD4^+^ T cells could indicate the possibility of previous exposures to common cold coronaviruses (HCoV, HCoV-OC43, HCoV-HKU1, HCoV-NL63, and HCoV-229E) that widely circulate and have sequence homology to SARS-CoV-2 (19, 23).

To formulate targeted public health strategies, understanding SARS-CoV-2 transmission risk factor among exposed close contacts of infected patients is important. Studies conducted for close contacts in China (24) and Singapore (25) have highlighted the importance of serological testing and limitation of RT-PCR testing for effective surveillance. Ng et al. have reported that among the close contacts considered for the study, symptom-based RT-PCR testing strategy missed 62% of COVID-19 diagnosis, and 36% of them were asymptomatic. Few studies have also focused on understanding the functional and phenotypic landscape of SARS-CoV-2-specific memory T-cell response in virus-exposed RT-PCR negative close contacts (26, 27). Wang et al. have reported the presence of SARS-CoV-2-specific CD4^+^ and CD8^+^ T memory cells in RT-PCR negative and seronegative close contacts, although their proportion was less than that of infected patients.

In India, a serosurvey conducted across 17 states and 2 union territories reported that the average seropositivity was 10.41% and approximately 75.3% of the seropositive individuals were asymptomatic (28). The authors have also mentioned that a follow-up after 3 or 5 months revealed a decline in seropositivity and neutralization activity among the cohort.

In this study, we explore the humoral and cellular immunity responses in the asymptomatic close contacts of COVID-19-positive patients.

## Materials and Methods

### Study Design

Last year during the period of June to July, large groups of migrant workers in India started returning to their native residence in shared transports. Our study cohort consisted of 47 migrant workers traveling from Gujarat to Odisha by bus. From the SARS-CoV-2 RT-PCR surveillance information of the migrant workers, we observed that some individuals have not contracted the disease even after traveling in close vicinity (proximity <1 m) of COVID-19-bearing individuals. To understand the immune status of these individuals (asymptomatic direct contacts <1 m proximity), we enrolled three subject groups for our study, namely, control (CTRL, unexposed to SARS-CoV-2), infected (INF, COVID-19 positive, inception confirmed by RT-PCR test), and asymptomatic contact (CON, individuals who traveled with SARS-CoV-2 RT-PCR positive individuals in the same vehicle sitting within ~1 m radius for 4 days). For ease of understanding, from herein, asymptomatic contacts will be designated as contacts. Blood samples from symptomatic infected individuals (n = 23) and contacts (n = 24) were collected after 10–11 days from COVID hospital or quarantine centers, respectively, with due approval from the concerned ethical committee. Among the infected individuals based on the disease symptoms on the day of sample collection, we further divided them into two subgroups, namely, symptomatic (SYM, showing mild or moderate symptoms) and asymptomatic (ASY). On the same day, these samples were received at ILS, Bhubaneswar, for isolating peripheral blood mononuclear cells (PBMCs) to mitigate technical variability. This study has considered candidates of both sexes with age ranging from 18 to 60 years. The control samples (14 individuals) were collected from healthy donors during the early days of pandemic when SARS-CoV-2 infections were not spread in Odisha state. They were further verified by RT-PCR negative results (in house SARS-CoV-2 testing facility following proper BSL-3 safety guidelines in ILS, BBSR), while they were never exposed to the SARS-CoV-2 antigen, evident from the SARS-CoV-2-specific surrogate virus neutralization serum antigen test.

### Enzyme-Linked Immunosorbent Assay

For IgA and IgM quantification, receptor binding domain (RBD) (SARS-CoV-2 spike RBD recombinant protein, mFc-Tag, CST # 41701S) antigen at a concentration of 200 ng/well was coated in a 96-well high binding microtiter plate (HIMEDIA-EP1) in 1× Tris-buffered saline (TBS) pH-7.4 for 2 h at 37°C. Following incubation, the plate was washed 3× times with a wash buffer (TBS containing 0.2% Tween 20). Blocking was done with milk (3% skim milk in TBS containing 0.05% Tween 20) for 1 h at 37°C. Thereafter, wells were washed, and 50 µl of serum (1:320 diluted) was added for 1 h at 37°C. Secondary antibody horseradish peroxidase (HRP) goat antihuman IgA (Biolegend #411002) and GtX Hu IgM HRP (Merck Millipore # Lot: 3462097) were used at a dilution of 1:2,500 for 1 h at 37°C for IgA and IgM quantification, respectively. Finally, after washing, 50 µl of 3,3′,5,5′-tetramethylbenzidine (TMB) substrate (Biolegend # 421101) was added for development of color for approximately 15 min. The reaction was stopped using 2N H_2_SO_4_, and absorbance was measured at 450 nm in a Multiskan reader (Thermo scientific).

For quantification of total COVID-19 and IgG antibodies, kits, namely, COVID-19 (IgG + Ig M + Ig A) Microlisa and Covid Kawach IgG Microlisa (# IR200196), were used (J. Mitra & Co.), respectively. The tests were performed according to the manufacturer’s recommended protocol.

### Bio-Plex

Human cytokine quantification was performed from serum samples according to manufacturer’s protocol (Bio-Plex Pro Human Cytokine Screening Panel #10000092045, Part no. 12007283). Briefly, 50 µl of 1× beads was added to the wells and washed two times with 200 µl of wash buffer. Fifty microliters of standards, samples (1:4 diluted), and controls were added and incubated on the shaker at 850 rpm for 30 min at room temperature (RT), following which in the same manner, 25 µl of 1× detection antibody was mixed and incubated. Thereafter, the wells were washed, and streptavidin-PE was added for 10 min with shaking. Eventually, after giving final washes, the samples were resuspended in 125 µl of assay buffer, and data acquisition was performed on Bio-Plex 200 System.

### Flow-Cytometry-Based Sorting of B and T Cells From PBMCs

PBMCs were isolated from unmatched control (n = 3), contact (n = 3), and SARS-CoV-2-infected individuals (n = 3). Cells were washed with Roswell Park Memorial Institute (RPMI)-1640 media containing 10% fetal bovine serum (FBS) and proceeded for staining in 1× fluorescence-activated cell sorting (FACS) buffer [3% FBS in 1× phosphate-buffered saline (PBS)]. Single-cell suspension was counted and blocked with Human TruStain FcX™ Fc Blocking reagent (BioLegend # 422302) for 10 min on ice. After blocking, cells were washed with 1× FACS buffer and stained with well-characterized TotalSeq-C antibodies for hash-tagging for effective pooling of samples afterwards (C0251 BioLegend # 394661, C0252 BioLegend # 394663, and C0253 BioLegend # 394665). Following hash-tagging, they were incubated with conjugated primary antibodies antihuman CD19 PB (eBiosciences # 48-0199-42) and antihuman CD3e FITC (eBiosciences # 35-0037-T100). Furthermore, they were washed and treated with PI viability dye (BioLegend # 79997) to proceed for sorting. CD3 and CD19 positive viable cells were collected in BD FACS melody cell sorter using a 70-µm sort nozzle. Overall, PBMCs from individual samples were counted; then, equal cells were hash-tagged followed by FACS sorting and eventually pooled for GEM preparation and single-cell RNA sequencing (scRNA-seq).

### Single-Cell Gene Expression and V(D)J Sequencing

The pooled and sorted single-cell suspensions were counted and encapsulated in 10× barcoded scRNA-seq gel beads with cDNA synthesis reagents using the 10× chromium instrument. Single-cell partitioning was performed using 10× Chromium Single-Cell G Chip kit followed by library preparation using Chromium Single-Cell 5′ Library kit according to 10× Genomics recommended protocol. Approximately 10,000 cells each for control, asymptomatic contacts, and infected groups were added to each channel, and cells recovered were 4,287, 8,924, and 7,558 respectively. Single-Cell V(D)J Enrichment kit was used to profile the human B- and T-cell receptors at single-cell level. The single-cell RNAseq libraries were then sequenced using an Illumina NextSeq550 platform.

### Virus Neutralization Assay

Neutralization assay was performed according to manufacturer’s protocol (SARS-CoV-2 Surrogate Virus Neutralization Test Kit, Genscript # L00847-A). Briefly, positive control, negative control and samples (serum 1:4 dilution) were diluted with HRP-RBD in a ratio of 1:1 in tubes and incubated at 37°C for 30 min. After incubation, 100 µl of each mixture was added to the plate for 15 min at 37°C, following which the wells were washed with 1× wash solution four times, and finally, TMB substrate was added for development of color. The reaction was stopped using 50 µl of stop solution, and absorbance was measured immediately in a Multiskan reader (Thermo Scientific). The percent inhibition/neutralization was calculated using the formula = (1 − OD value of sample/OD value of negative control) × 100%.

### scRNA-seq Data Processing

The Cell Ranger pipeline (v5) from 10× Genomics was used to demultiplex raw BCL files to fastq files and subsequently map the reads to the human reference genome (refdata-cellranger-GRCh38-3.0.0). 10× feature barcode information was included in the feature reference file and was passed onto the cell ranger multi-command. The resulting output generated was a gene expression matrix along with feature barcode counts for each cell barcode. These matrices were then processed in R (v3.5) using the Seurat package (v3.2.3) (29). As a primary quality control (QC) step, we first filtered out cells that were captured with <300 expressed genes. Next, for filtering out potential doublets, we have discarded cells with a total number of detected genes >1,800. Furthermore, we have calculated the percentage of mitochondrial (MT) genes expressed and discarded cells with more than 5% mitochondrial genes of all the detected cells. The QC steps were performed for each subject group separately. After integrating all the samples and performing dimension reduction analysis, clusters that had enriched ribosomal (RPS and RPL) genes were not considered for further analysis. Furthermore, after annotating the clusters, B cells that expressed *CD3E* (average expression >1) and T cells that expressed *MS4A1* (average expression >1) were removed from the analysis, thus yielding a total of 7,848 cells.

The feature barcoded samples were demultiplexed using Seurat’s multimodal analysis. Individual samples were normalized for cell counts using Sctransform (30) method in Seurat.

### Sample Integration

To remove batch effects, the individual normalized samples were integrated using the integration method implemented in Seurat.

### Dimension Reduction and Clustering

For the integrated object, principal component analysis (PCA) was performed (npcs = 30) to reduce the dimensionality of the dataset. Thirty principal components were then used to compute the k-nearest neighbor graph, which was further used to find clusters. We visualized the clusters by UMAP. We checked for marker genes in each cluster using the function FindAllMArkers (min.pct = 0.25 and logfc. threshold = 0.38). We used the default non-parametric Wilcoxon rank-sum test for the differential expression analysis. Clusters that had enriched ribosomal (RPS and RPL) genes were regarded as low-quality clusters and discarded from the analysis, further performing the above-mentioned dimension reduction and clustering analysis again.

### Cell-Type Annotation

The function FindAllMarkers was used to find out the marker genes in each of the clusters. Average expression and DoHeatmap functions were used to visualize the expression of the top marker genes in each cell cluster. These top markers and other known canonical markers were used to determine the cell type of each cluster. Differential analysis of selected clusters was performed using Find Markers function (min.pct = 0.25 and logfc. threshold = 0.38).

### TCR and BCR Data Integration

TCR-seq and BCR-seq data were assembled using Cell Ranger pipeline (v5, 10× Genomics) with the cell ranger multi-command using the reference genome (refdata-cellranger-vdj-GRCh38-atlas-ensembl-3.10). For each of the samples, the output file, filtered_contig_annotations.csv, containing TCR-α chain and TCR-β chain CDR3 nucleotide sequences for single cells were generated. Using a custom code (https://www.biostars.org/p/383217/), the V(D)J information from filtered_contig_annotations.csv was added into the metadata of Seurat object for individual samples.

### Subclustering of T/NKT/NK and B Cells

For in-depth analysis of T and B clusters, we selected these clusters from the integrated Seurat object by using the function subset. Cells expressing both TCR and BCR sequences were removed from the analysis. Reclustering analysis was performed as mentioned above. A detailed cell-type annotation was obtained by exploring the top marker genes along with the canonical genes for T and B cells, respectively. Differential gene expression analysis was performed as mentioned above.

### Functional Enrichment Analysis

Gene Ontology and Kyoto Encyclopedia of Genes and Genomes (KEGG) pathway analyses were performed in R using the package ClusterProfiler (31). This package supports statistical analysis and visualization of functional profiles for genes and gene clusters.

### TCR-seq and BCR-seq Analysis

The R package ScRepertoire (v1.2.1) (32) was used to combine the contig annotation data from filtered_contig_annotations.csv of three samples to a single list object. This was done by using the functions combineTCR and combineBCR. The combined TCR and BCR contig list files were then integrated with the corresponding Seurat object of the scRNA-seq data using the function combine expression with default parameters. Only the cells with both TCR/BCR and scRNA-seq data were kept for downstream clonotype analysis.

### Statistical Tests

All the statistical tests are performed in R, and the bar and box plots are generated using the GGPUBR package. The pie chart of antibodies was created using the GGPLOT2 package, and all the respective p-values in box plots are calculated using Wilcoxon test and stat_compare_means (paired = FALSE) function for respective condition pairs.

## Results

### Antibody Responses to SARS-CoV-2 With Neutralizing Magnitude

**Figure 1:**
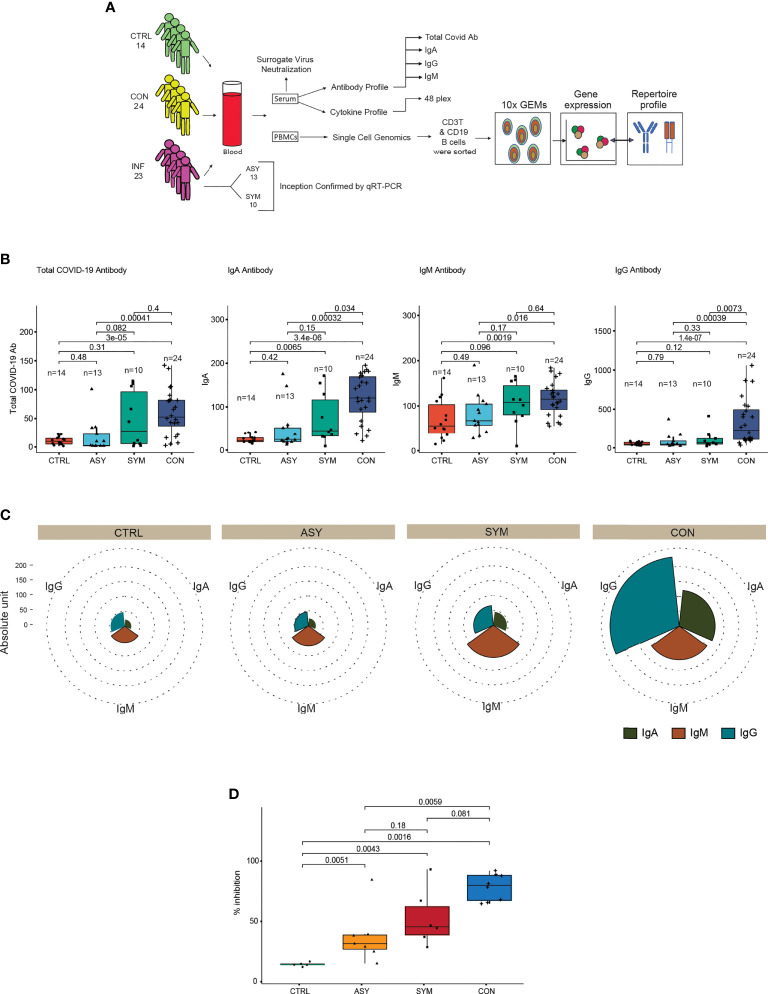
Study approach and distinct antibody responses to SARS-CoV-2 along with neutralization assay. **(A)** Outline delineating the experimental workflow. **(B)** Box plots showing the anti-SARS-CoV-2 total, IgA, IgM, and IgG antibody proportions in serum samples from controls, symptomatic- and asymptomatic-infected individuals and in contacts **(C)**. Pie chart showing the overall immunoglobulin response across subject groups. **(D)** Box plot depicting the percent inhibition of neutralizing antibodies in an *in vitro* spike RBD-ACE2 interaction-based surrogate virus neutralization assay. Statistical comparisons were performed using unpaired Wilcoxon test. p-values are written against respective comparisons, where n = number of individuals; IgA, immunoglobulin A; IgM, immunoglobulin M; IgG, immunoglobulin G; control (n = 14), infected (n = 23), symptomatic (n = 10), asymptomatic (n = 13), and contact (n = 24).

We assessed our data consisting of 61 individuals, 18 females and 43 males, distributed into three groups: control (n = 14), infected (n = 23), and contact (n = 24) (**Supplementary Figure 1A**). The characteristics of these subject groups are provided in the supplementary information (**Supplementary Table 1**). The study design and methodology used has been depicted in detail (**Figure 1A**). We performed ELISA from serum samples to quantify total and subtype antibodies specific to spike receptor binding domain (RBD). Upon groupwise comparison of the mean levels of total, IgA, IgM, and IgG neutralizing antibodies (**Figure 1B**), we observed contact individuals to have significantly high levels of IgA and IgG antibody titers, IgA being indicative of neutralizing respiratory viruses and protecting mucosal surfaces by hindering their attachment to epithelial cells (14). On the other hand, significant amounts of IgG and a higher level of IgM dictate responsiveness towards SARS-CoV-2. Categorization of the infected patients depicted (**Figure 1B**) symptomatic individuals to have elevated levels of IgA, IgM, and IgG in comparison to asymptomatic patients in the median level although the changes were not significant. The overall antibody distribution of IgG and IgA was higher in contacts compared to control, symptomatic, and asymptomatic individuals. The median levels of IgM were similar in contacts and symptomatic individuals but lower in controls and asymptomatic (**Figure 1C**). Furthermore, to validate the COVID-19-neutralizing efficacy of the serum samples, we performed an *in vitro* spike RBD-ACE2 interaction-based surrogate virus neutralization assay of some representative (**Supplementary Table 1**) samples. Neutralizing antibodies against SARS-CoV-2 were detected in serum samples of contact, symptomatic and asymptomatic individuals in gradient (**Figure 1D**). The percent inhibition shown by contact individuals in accordance to control and infected individuals are noteworthy and correlate with their high neutralizing antibody titers.

### Serum Cytokine Levels With Contrast of Differentiation Across Subject Groups

**Figure 2:**
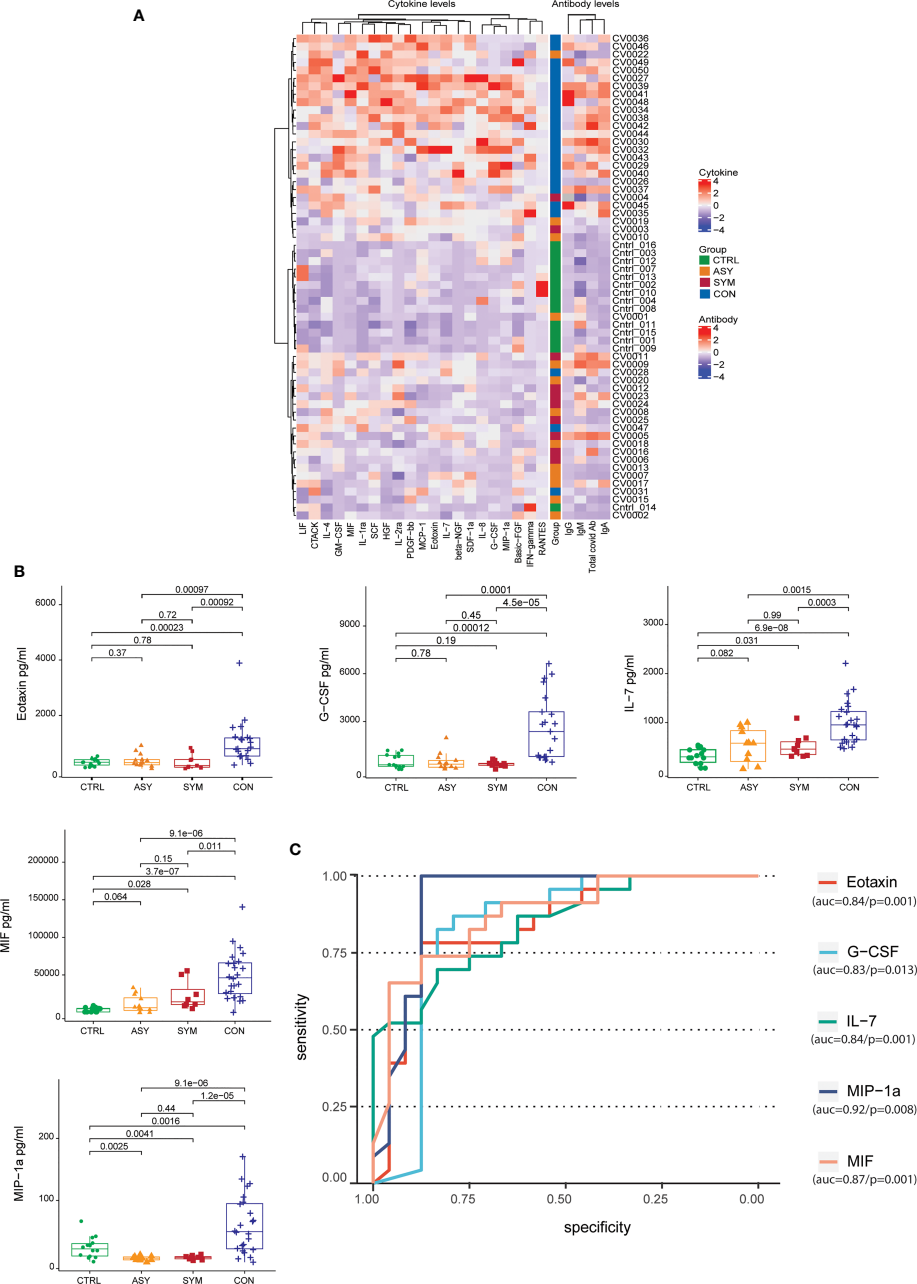
Unsupervised hierarchical clustering of antibodies and cytokines and cytokine levels with contrast of differentiation across subject groups. **(A)** Heatmap depicting the grouping (row-wise clustering) of individuals based on the abundance of 21 cytokines in serum and the respective antibody levels. The column-wise clustering portrays the overall coexpression of cytokines. Both row-wise clustering and column-wise hierarchical clustering were performed using Ward D method with 500 bootstraps. **(B)** Box plots depicting the serum cytokine levels in pg/ml (Eotaxin, G-CSF, IL-7, MIF, and MIP-1α) determined by Bio-Plex along with the **(C)** the area under the receiver-operating characteristic (ROC) curve of the cytokines having more than 0.8 AUC, taking 14 controls, 10 symptomatic, and 13 asymptomatic patients along with 24 contacts. Statistical comparisons were performed using unpaired Wilcoxon test; p-values are written against respective comparisons.

Furthermore, to complement our understanding about the nature of protection observed in contact individuals, we clustered the subject groups based on cytokine levels. Correlating the antibody profile with the cytokine data by unsupervised hierarchical clustering (number of bootstraps = 500), we observed three clusters. Contacts and controls formed two distinct clusters and a third cluster representing a mixed cytokine and antibody profile for some of the contact and infected individuals (**Figure 2A**). We found the cytokine levels to be differentially secreted between contact and infected individuals. To associate cytokine levels with clinical consequences, we performed logistic regression analysis [Pr(>|z|) < 0.05], using scaled cytokine levels and denoted the outcomes as binary variables (contact as 1 and infected as 0). We observed that out of 48 cytokines, 21 were able to distinguish between the two groups, among which 5 cytokines were showing prominent distinction, namely, Eotaxin, granulocyte-colony stimulating factor (G-CSF), IL-7, migration inhibitory factor (MIF), and macrophage inflammatory protein-1α (MIP-1α), and the secretory levels were highly significant in contacts with respect to infected and control individuals (**Figure 2B**). The area under the curve of the receiver operating characteristic (ROC) line had more than 0.80 area under the curve (AUC) (**Figure 2C**), which suggested an aggregated measure of performance between contact and infected individuals. The other 16 cytokines, namely, CTACK, basic FGF, GM-CSF, HGF, IFN-gamma, IL-1ra, IL-2ra, IL-4, IL-8, LIF, MCP-1, beta-NGF, PDGF-bb, RANTES, SCF, and SDF-1α, also showed a higher trend in contact individuals (**Supplementary Figure 1B**) but reduced contrast. This analysis further strengthened our observation and gave an insight into the cytokine responses in direct contacts and infected patients. A combination of this handful of cytokines with elevated levels of IgA and IgG might play pivotal roles in evading viral infections and can be contemplated as protective signatures.

### Single-Cell Characterization of B and T Cells From PBMCs

**Figure 3:**
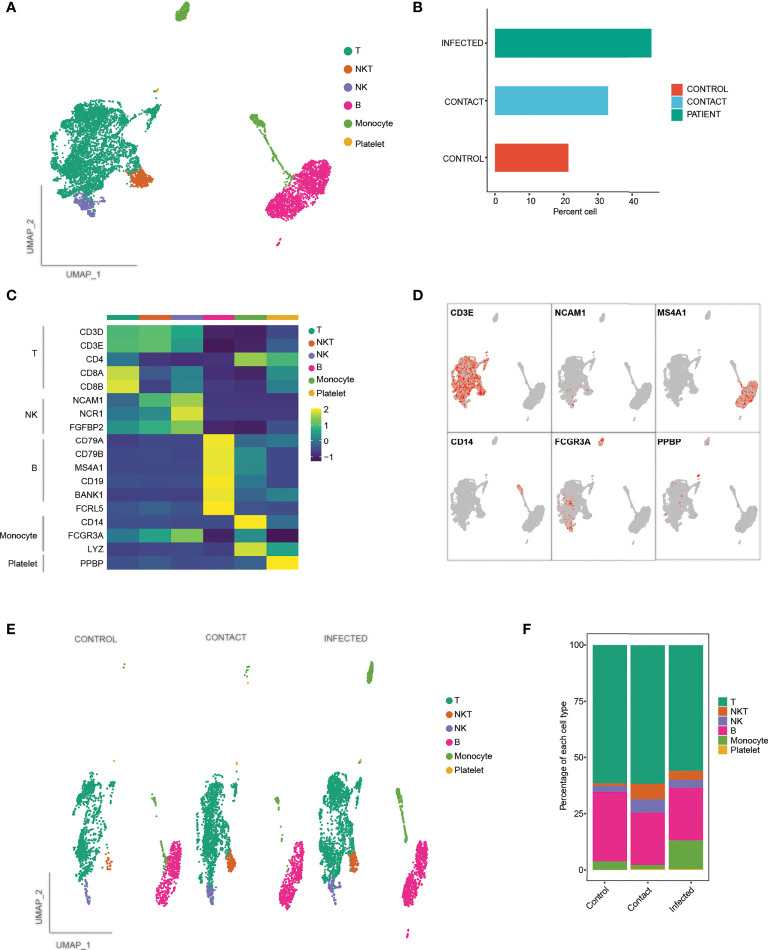
scRNA-seq analysis and demarcation of cell types according to characteristic markers. **(A)** UMAP representation of 7,848 single cells, color coded according to cell type. **(B)** Bar plot showing the percentage of cells contributed by control, contact, and infected samples. **(C)** Heat map showing canonical markers for individual T, B, NK, NKT, monocytes, and platelets, respectively. **(D)** UMAP representation of canonical markers for T cells (CD3E), B cells (MS4A1), NK (NCAM1, FCGR3A), NKT (CD3E, NCAM1), monocytes (CD14, FCGR3A), and platelets (PPBP). **(E)** UMAP representation of clusters stratified per individual samples: controls, contact, and patients color coded according to cell types. **(F)** Bar plot showing relative contribution of each of the cell types in controls, contact, and patients.

Furthermore, to understand the cellular basis of protective immune signatures, we characterized the immunological features of contacts in detail. The sequencing depth for each of the sample is provided in **Supplementary Table 1**. We first sorted CD3^+^ and CD19^+^ cells by flow cytometer to select T and B lymphocyte populations, respectively (**Supplementary Figure 2A**) from PBMCs and performed scRNA-seq along with paired B-cell and T-cell repertoire profiling at single-cell resolution. We selected three contacts, three COVID-19-infected patients, and three healthy controls for this analysis. After passing the quality control parameters (see *Materials and Methods* for details), we obtained a total of 7,848 cells, comprising 1,679 cells (21.3%) from control, 2,589 cells (32.9%) from contacts, and 3,580 (45.6%) cells from patients (**Figure 3B**). Dimension reduction and unbiased clustering analysis identified 19 clusters, which were further categorized into six cell types according to their canonical markers (**Figures 3A, C, D**; **Supplementary Figure 2C**). In addition to majority of T (n = 4,640) and B (n = 1,956) lymphocytes, we have also obtained monocytes (n = 568), natural killer (NK) (n = 323) cells, natural killer T (NKT) (n = 341) cells, and platelets (n = 20). There was no cluster bias among the samples (**Supplementary Figure 2B**). Interestingly, we did not observe much difference in cell-type compositions among the samples except for monocytes, which were found to be enriched in patients compared to control and contacts (**Figures 3E, F**). Although CD14^+^ and platelets were not considered for our study, re-evaluation of post-sorted cells revealed CD14 signatures, since differences in purity of samples can be a plausible reason for monocyte contamination. All the three infected patients considered for scRNA sequencing showed disproportionate fractions of CD14^+^ cells, since the purity of CD3^+^ cells after sorting was approximately 82%. For in-depth analysis of individual cell clusters, we had dropped a control sample (C2) from the analysis due to the presence of enriched B cells in the individual compared to other control individuals (**Supplementary Figure 2D**). We followed the second control individual and found that she had a long history of asthma and skin allergy, which might be the reasons behind higher percentages of B cells.

### Phenotypic Characteristics of T, NK, and NKT Cells

**Figure 4:**
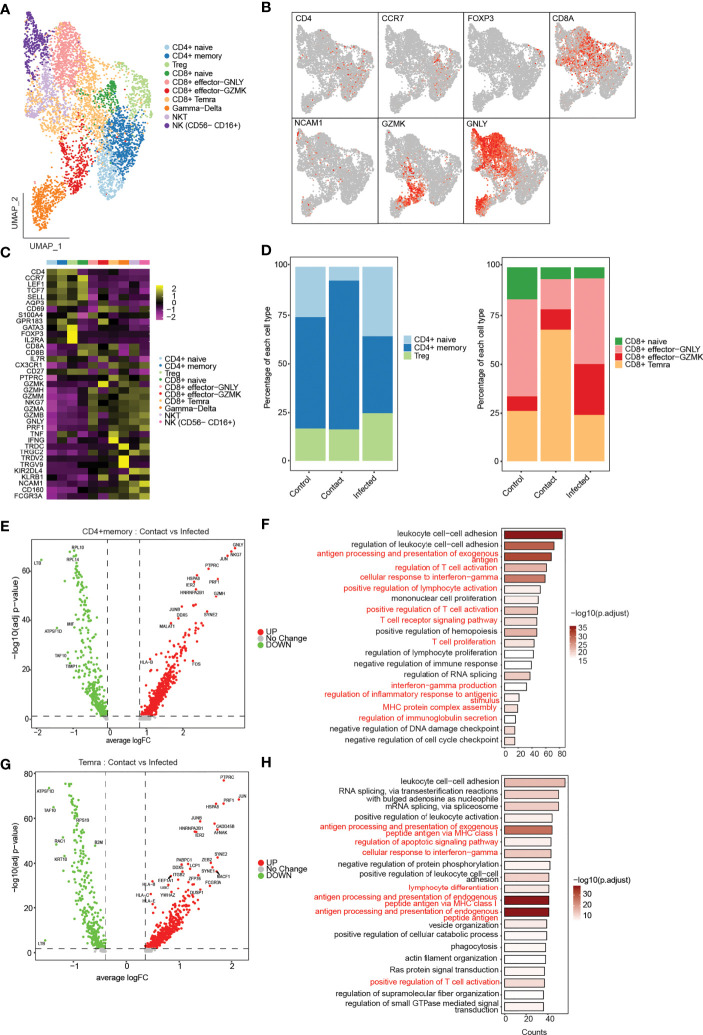
Phenotypical characterization of T, NK, and NKT cells. **(A)** UMAP representation of 5,130 single cells, color coded according to T-cell subtypes. **(B)** UMAP representation of subtype-specific marker genes: *CD4, CCR7, FOXP3, C8A, NCAM1, GZMK, and GNLY*. **(C)** Heatmap showing expression of marker genes for CD4^+^ naive (*CCR7^−^, LEF1, TCF7, SELL*), CD4 memory (*AQP3, CD69*), Treg (*FOXP3, IL2RA*), CD8 naive (*CCR7, LEF1, TCF7, SELL*), CD8^+^ effector GNLY (*GNLY, GZMH, NKG7*), effector GZMK (*GZMK*), CD8^+^ Temra (*PTPRC, CX3CR1*), gamma–delta (*TRDV2, TRDC, TRGV9*), NKT (*NCAM1, KLRB1*), and NK (*NCAM1^−^ FCGR3A^+^*). **(D)** Bar plot showing relative contribution of the three subclusters of CD4^+^ T cells (left), and four subclusters of CD8^+^ T cells in controls, contact, and patients (right). **(E)** Volcano plot showing the differentially expressed genes of CD4^+^ memory T cells between the contacts and infected patients. p-values were calculated using non-parametric Wilcoxon rank-sum test, and adjusted p-value were calculated based on the Bonferroni correction procedure. **(F)** GO BP enrichment analysis of differentially expressed genes upregulated in CD4^+^ memory T cells in contacts. p-values were calculated using non-parametric Wilcoxon rank-sum test, and adjusted p-value were calculated based on the Bonferroni correction procedure. **(G)** Volcano plot showing the differentially expressed genes of CD8^+^ Temra T cells between the contacts and infected patients. **(H)** GO BP enrichment analysis of differentially expressed genes upregulated in CD8^+^ Temra T cells in contacts. p-values were calculated using non-parametric Wilcoxon rank-sum test, and adjusted p-values were calculated based on the Bonferroni correction procedure.

A total of 5,134 of T, NK, and NKT cells were recovered from clusters depicting 10 different phenotypes (**Figure 4A**). According to the canonical markers, we identified three subtypes of CD4^+^ T cells (*CD3E* ^+^, *CD4* ^+^), CD4^+^ naive (*CCR7* ^+^, *LEF1* ^+^, *TCF7* ^+^, *SELL* ^+^), CD4^+^ memory (*AQP3* ^+^, *CD69* ^+^), and Treg (*FOXP3* ^+^, *IL2RA* ^+^) (**Figures 4B, C**). CD4^+^ memory cells showed high expression of *GATA3*, thereby implying that they were type-2 helper T (TH2) cells (**Figure 4C**). CD8^+^ T cells (*CD3E* ^+^, *CD8A* ^+^, *CD8B* ^+^) were divided into four subtypes: CD8^+^ naive (*CCR7* ^+^, *LEF1* ^+^, *TCF7* ^+^, *SELL* ^+^) and CD8^+^ effector cells (*S100A4* ^+^, *GPR183* ^+^), which were further subdivided into CD8^+^ effector GNLY, which expressed cytotoxic genes *GNLY*, *GZMH*/*M*/*A*/*B*, and *NKG7*, and CD8^+^ effector GZMK, which exclusively expressed *GZMK* (**Figures 4B, C**) but had low expression of other cytotoxic genes; and CD8^+^ Temra, which expressed *PTPRC* and *CX3CR1* along with cytotoxic genes *GZMH*, *PRF1*, *TNF*, and *IFNG*. Gamma–delta cells showed expression of their characteristic signature genes *TRDV2*, *TRDC*, *TRGV9*, and *KLRB1*. Cells expressing *CD8A* and *NCAM1* were classified as NKT cells. NK cells (*NCAM1* ^+^, *NCR1* ^+^) that showed high expression of *FCGR3A* and low expression of *NCAM1* were classified as NK (*NCAM1* ^−^ *FCGR3A* ^+^) (**Figures 4B, C**).

Next, to assess the prevalence of each of the T-cell subtypes, we compared the cell-type composition across three conditions (**Supplementary Figures 3A, B**). The composition of T, NKT, and NK cells differed among the three groups. In asymptomatic contacts, the percentage of CD4^+^ T cells were decreased, whereas CD8^+^ T cells were increased compared to controls and infected patients (**Supplementary Figure 3C**). Interestingly, we also observed an increase in NKT cells in controls particularly in C1 compared to contacts and infected patients (**Supplementary Figures 3B, C**). Among CD4^+^ T cells, the percentage of naive cells decreased in contacts compared to controls and infected patients, whereas the fraction of memory cells was found to be increased in contacts (**Figure 4D**). This decrease in naive cells indicates the possibility of a previous exposure to some infection in contacts. Differential gene expression analysis of CD4^+^ memory T cells between contacts and infected patients revealed genes such as *CD45*, *CCL5*, *KLRK1*, *TNF*, *HLA-B*, *JUN*, and *FOS*, which are known to be involved in T-cell proliferation and activation, response to interferon-gamma production and antigen processing and presentation processes, were found to be significantly upregulated in contacts (**Figures 4E, F**). Pathway analysis revealed pathways related to TCR signaling, Th1, Th2, and Th17 cell differentiation, indicating the ability of rapid effector response of these cells (**Supplementary Figure 3D**). Among CD8^+^ T cells, CD8^+^ effector GNLY and CD8^+^ effector GZMK were decreased in contacts compared to that in patients. In contrast, we observed CD8^+^ Temra cells to be drastically increased in contacts (**Figure 4C**). CD8^+^ Temra cells are terminally differentiated effector cells, which exhibit potent effector functions by secreting proinflammatory cytokines and chemokines. Differentially expressed genes, such as *CCL5*, *KLRK1*, *TNF*, *IFNG*, *PRF1*, *JUN*, *JUNB*, and *HLA-B*, were upregulated in Temra cells of contacts in comparison to infected patients (**Figures 4G, H**). These genes are known to be involved in processes such as cellular response to IFN-γ, inflammation, antigen processing and presentation, T-cell activation, and TCR signaling (**Figure 4H** **;** **Supplementary Figure 3E**).

### Expanded TCR Clones and Usage of V(D)J Genes

**Figure 5:**
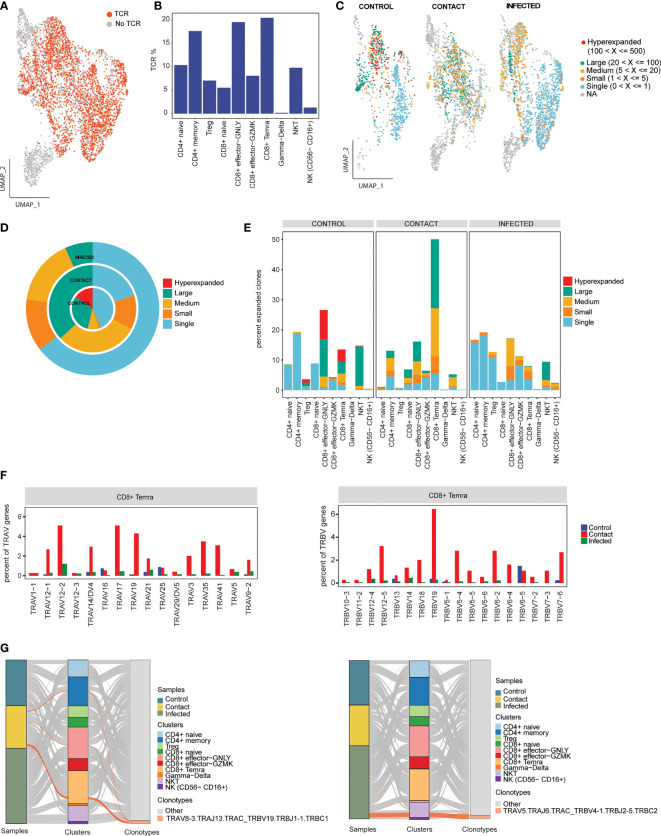
Clonal expansion of T-cell receptors. **(A)** UMAP representation of T/NK/NKT cells colored by TCR detection. **(B)** Bar plot showing percentage of TCR detected in each cell cluster. **(C)** UMAP representation of T/NK/NKT cells colored by their clonal expansion status. Clones were defined as single (n = 1), small (1 < n ≤ 5), medium (5 < n ≤ 20), large (20 < n ≤100), and hyperexpanded (100 < n ≤ 500). **(D)** Nested pie plots showing the clonal status across controls, contacts, and infected patients. **(E)** Bar plot showing percentage of expanded clonotypes in each cluster across samples. **(F)** Bar plots showing usage of some TRA genes (left) and TRB genes (right) in CD8^+^ Temra cell-type across subject groups. **(G)** Alluvial plots showing relationship of the top V(D)J pairing frequencies of expanded clonotypes for contacts (left) and infected patient (right) between samples and cell clusters.

T-cell receptor is important for antigen recognition and T-cell-mediated virus clearance. To understand the TCR bias across controls, contacts, and infected samples, we reconstructed the TCR sequences from single-cell TCR sequencing (**Figure 5A**). We found that CD4^+^ memory, CD8^+^ effector GNLY, and CD8^+^ Temra cell types had higher percentages of matched TCR sequences compared to the other subtypes (**Figure 5B**). When compared across samples, we found that CD8^+^ Temra cells had the highest percentage of matched TCR in contacts (50%) compared to the infected individuals and controls (**Supplementary Figures 3F, G**). TCR clonal expansion was profound in asymptomatic contacts and infected patients compared to controls, owing to viral exposures. On the contrary, when we compared contacts with patients, the extent of clonal expansion was found to be more in contacts (~44% more than infected) (**Figures 5C, D**). However, we also observed hyperexpanded clonotypes in one of the control samples (**Supplementary Figure 3H**). Different degrees of clonal expansion were observed among the cell types (**Figure 5E**). In contacts, CD8^+^ Temra cells showed a high percentage of clonally expanded cells (~45%) (**Figures 5C, E**), suggesting that these cells had undergone dynamic state transitions.

Furthermore, we explored gene preference of TCRs across controls, contacts, and infected individuals. We observed overrepresentation of TRAV (*TRAV17, TRAV12-1, TRAV19, TRAV35, and TRAV41*) and TRBV (*TRBV12-5 and TRBV19*) genes in contacts compared to patients (**Figure 5F** **;** **Supplementary Figure 3I**). The top V(D)J pairing frequency in expanded clones was found to be *TRAV8-3. TRAJ13.TRAC_TRBV19.TRBJ1-1.TRBC1* (n = 59), which contributes to a major proportion of the clonotypes in CD8^+^ Temra subset in contacts, whereas in patients, it was found to be TRAV5.TRAJ*6. TRAC_TRBV4-1.TRBJ2-5.TRBC2* (n = 89) contributes to a major proportion of the clonotypes in the NKT subset. In control, *TRAV8-3. TRAJ43.TRAC_TRBV3-1.TRBJ2-2.TRBD1.TRBC2* (n = 123) contributed majorly to CD8^+^ effector GNLY cells (**Figure 5G**; **Supplementary Figure 5A**).

### Characterization of B Cells and Clonal Expansion of B-Cell Repertoire

**Figure 6:**
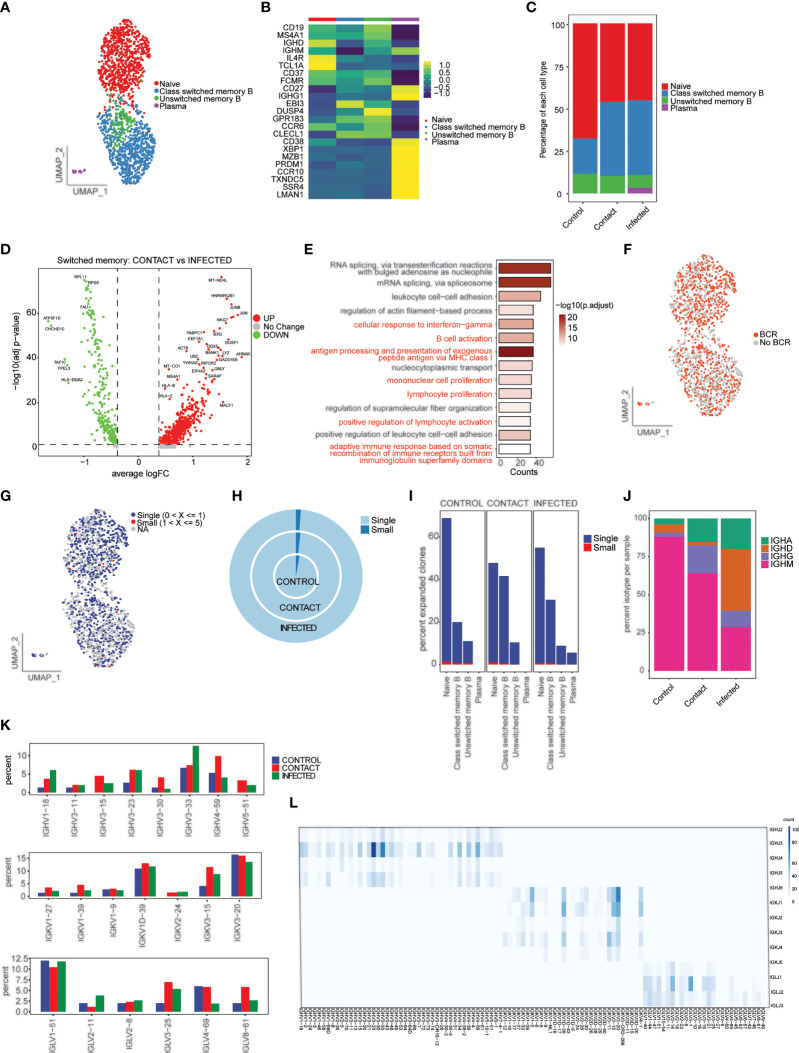
Characterization of B cells and B-cell repertoire. **(A)** UMAP representation of 1,626 single cells, color coded according to B-cell subtypes. **(B)** Heatmap showing expression of marker genes for naive B (*CD19^+^ IGD^+^ CD27^−^*), class switched memory B (*CD19^+^ IGD^−^ CD27^+^*), unswitched memory B (*CD19^+^ IGD^+^ CD27^+^*), and plasma (*CD38, XBP1, MZB1*). **(C)** Bar plot showing relative contribution of B-cell subtypes in control, contact, and infected patient samples. **(D)** Volcano plot showing the differentially expressed genes of class switched memory B cells between the contacts and infected patients. p-values were calculated using non-parametric Wilcoxon rank-sum test, and adjusted p-value were calculated based on the Bonferroni correction procedure. **(E)** GO BP enrichment analysis of differentially expressed genes upregulated in class switched memory B cells in contacts. **(F)** UMAP representation of B cells colored by BCR detection. **(G)** UMAP representation of B cells colored by their clonal expansion status. Clones were defined as single (n = 1), small (1 < n ≤ 5). **(H)** Nested pie plots showing the clonal status across samples. **(I)** Bar plot showing percentage of expanded clonotypes in each cluster in subject groups. **(J)** Bar plot showing percentage of IGHA, IGHM, IGHG, and IGHD isotypes in control, contact, and infected patient samples. **(K)** Bar plot showing usage of some IGH genes (top), IGK genes (middle), and IGL (bottom) genes **(L)**. Heatmap showing IGH/K/L rearrangement in contact. Colors indicate the frequency of specific gene pairs.

A total of 1,626 single cells were recovered, which were clustered into four phenotypes: naive B cells (*CD19^+^ IGD^+^ CD27^−^*), class switched memory B cells (*CD19^+^ IGD^−^ CD27^+^*), unswitched memory B cells (*CD19^+^ IGD^+^ CD27^+^*), and plasma cells (*CD38*, *XBP1*, *MZB1*) (**Figures 6A, B**). We did not observe much difference in cell-type composition between contact and patients (**Figure 6C**; **Supplementary Figures 4A, B**). Class switched memory B cells produce long-lived plasma cells and memory B cells capable of responding to secondary challenges (33). Differential gene expression analysis in class switched memory B cells between contact and infected patients revealed genes that are involved in B-cell activation, response to interferon-gamma, adaptive immune response based on somatic recombination, etc. to be significantly upregulated in contacts (**Figures 6D** **, E** **;** **Supplementary Figure 4C**).

To understand the BCR bias among subject groups, we reconstructed the BCR sequences (**Figure 6F**). Naive B and class switched memory B cell types had highest percentages of matched BCR sequences (**Supplementary Figure 4D**). When compared across samples, we found that class switched memory B cells had the highest percentage of matched BCR in contacts (42%) compared to patients and controls (**Supplementary Figures 4E, F**). We did not find much clonal expansion across the three samples (**Figures 6G** **–I**). We also observed overrepresentation of IgG isotype in contacts (18.5%) compared to patients and controls (**Figure 6J**; **Supplementary Figure 4G**). This result corresponds to previous observations in close contacts of COVID-19 patients (34) and to our antibody profile data.

Next, we studied the gene preference of BCRs in contacts, infected patients, and controls. We have observed overrepresentation of *IGHV4-59* and *IGHV3-15* in contacts. The preferred IGKVs and IGLVs were *IGKV1-1*, *IGLV3-27*, and *IGLV8-61*, respectively (**Figure 6K**; **Supplementary Figure 4H**). The top pairing frequency in contacts is *IGHV3-23-IGHJ4* (observed 105 times) (**Figure 6L**), whereas in control and patient samples, the top pairing frequencies are *IGHV3-33-IGHJ4* (observed 69 times) and *IGHV4-34-IGHJ4* (observed 170 times), respectively (**Supplementary Figures 5B, C**).

## Discussion

The nature of protection observed in contact individuals is of keen interest and of great concern keeping in view the recent scenario. The surged level of antibodies in contacts hints towards prior exposure or recovery from other pathogens having similar antigenic determinants. Increased levels of secretory IgA are efficient in neutralizing respiratory viruses. IgA may also be a broader cross-reactive antibody against various human coronaviruses as reported by Wec AZ et al. (35). On the other hand, significant levels of IgG are generally observed in COVID-19-infected individuals after 7–10 days and maintained at high levels for a longer period of time (36). This might be true for contact individuals, but its certainty needs to be ascertained. The reported levels of cytokine among mild, severe, and fatal COVID-19 patients differ drastically (37).

Of the 48 cytokines that we analyzed, 5 showed prominent contrast of differentiation among the contact individuals. Eotaxin, G-CSF, IL-7, MIP-1α, and MIF are found to be secreted across all subject groups and in significantly higher quantities in contacts than in others. MIF is a signature cytokine for increased disease severity (38), but in our case, its biology is fascinating to note. Among others, IL-7 and G-CSF are known to play pivotal roles in lymphocyte expansion and render protection against invading pathogens, respectively (39). It is also compelling to find that when we correlated the antibody profile with the cytokine data, contacts showing elevated levels of antibodies were also showing high levels of particular cytokines. Thus, the co-occurrence of a handful of cytokines with elevated Ig levels appeared to be a protective signature among contacts.

Along with antibody and cytokine responses, B and T cells also show effective immunity against SARS-CoV-2. Adaptive immune system recognizes pathogens and remembers them through humoral and cellular responses. These responses can either work in synergy or redundantly. Merely the presence or absence of antibodies does not correlate with immune memory (40). Our single-cell data fetched specific clusters of T and B cells along with minor populations of NK, NKT, monocytes, and platelets. Using scRNA-seq along with paired BCR and TCR profiling data, we have shown the immune profile of asymptomatic close contacts compared to COVID-19 patients. Although we found many similarities between the close contacts and patients, there are key immune differences that might be beneficial and help the contacts in resisting the virus infection.

Severe COVID-19 patients experience hyperactivation of immune responses, predominantly polyfunctionality in CD8^+^ T cells, distinct CD4^+^ T-cell subpopulations, and B-cell heterogeneity (41). In the case of SARS-CoV-2, there are reports that T cells may have long-term memory compared to B cells (20). From our study, we find the contacts to have lower fraction of CD4^+^ naive cells and higher fraction of CD4^+^ memory cells compared to infected patients, suggesting that initiation of immunity and generation of T-cell responses to SARS-CoV-2 can be mediated by possible exposure to the virus even in the absence of a successful infection. Wang et al. (26) showed the expansion of memory T cells to be superior and greater in COVID-19-positive individuals. Nevertheless, close contacts were also famed with preferable proportions of T cells of ample size, standard quality, and eventful proliferating capacity.

Virus-infected host cells process antigens through the endogenous pathway and lead to the formation of CD8^+^ responses (42). In all probability, the magnitude of CD8^+^ T-cell responses diminished due to insufficiency of viral antigens within the host cells of close contacts, but it increased the frequency of CD8^+^ T-cell memory to a greater extent. Conversely, CD4^+^ T-cell memory is not generated during endogenous processing of viral antigens but during phagocytosis of soluble viral particles and thus led to the plausible explanation of reduction in frequency of naive CD4^+^ T cells and increased CD4^+^ T-cell memory in contact individuals along with CD8^+^ Temra cells.

Another possibility is the function of cross-reactive clones that were probably acquired during previous infections with HCoVs (26). HCoVs are ubiquitous and account for 20% of “common cold” infections (22, 43). Reports on cross-reactivity suggesting T cells of individuals who gain protection against SARS-CoV-2 due to cross-reactivity are of clinical importance (6). The significance of T-cell cross-reactivity has been cited through the H1N1 pandemic (44). In this study, the authors have reported that CD8^+^ T cells endow protective immunity in viral outbreaks. Furthermore, the subset CD45RA^+^ CCR7^−^, also termed as late effector population, was found to confer this protection in H1N1 pandemic. In a review article, the authors have articulated that the possible reason for low incidence of COVID-19 cases in India till late March is due to the presence of cross-reactive immunity from previous exposures to human endemic corona viruses (45).

The CD4^+^ memory cells expressed genes enriched in T-cell activation, TCR signaling, and Th1, Th2, and Th17 differentiation pathways, indicating a rapid cellular response in contacts. Viral infections can be efficiently mitigated by cytotoxic lymphocytes like CD8^+^ T cells, NK cells, and NKT cells. CD8^+^ T cells can also undergo hyperactivated states and acquire functions related to NK and NKT cells (46). Contact individuals exhibited a higher percentage of CD8^+^ Temra subtypes, which are known to be highly cytopathic and are potent producers of IFN-γ (47). Moreover, TCR sequencing identified expanded T-cell clonotypes in contacts majorly in CD8^+^ Temra subtype.

We have also performed a detailed analysis of B cells and identified a higher percentage of class switched memory B cells in contacts. Memory B cells mount long-term protection against viruses. Specific pathways pertaining to B-cell activation, lymphocyte proliferation, and somatic recombination of immune receptors were significantly enriched in contacts. We do find increased IgG isotype in contacts, which goes hand in hand with our antibody profiling data. BCR sequencing did not reveal much difference in the frequency of expanded clonotypes among the samples, but we find biased gene usage and V(D)J recombination between the samples. We report the strongest pairing frequency in contact to be IGHV3-23-IGHJ4, which was previously reported in infected patients (11). Overall high antibody titers, its neutralizing capacity, selective cytokine secretion, enriched CD4^+^ memory, CD8^+^ Temra, and class switched memory B cells may have bestowed asymptomatic contact individuals with protective immune signatures to steer clear of SARS-CoV-2 infection.

## Limitations of the Study

The information about any prior exposure of contact cohorts to other SARS viruses or asymptomatic SARS-CoV-2 infection could have been beneficial for the study. We also did not manage to correlate Bio-Plex cytokine profiling to our single-cell gene expression data, since most of the cytokine genes were missed out due to our stringent quality control of data. We emphasized on the gene expression and TCR profiles of CD3^+^ T and CD19^+^ B lymphocytes for delineating the specific protective features against SARS-CoV-2 leaving behind other cellular markers like plasma B-cell subtypes, which could have strengthened our study further. The plasma B-cell subtype characterization and functionality would have been relevant to associate our antibody profiling data.

## Data Availability Statement

The datasets presented in this study can be found in online repositories. The accession number for the data submitted in ArrayExpress is E-MTAB-10636.

## Ethics Statement

The studies involving human participants were reviewed and approved by Institutional Human Ethical Committee (Ref. no. 109/HEC/21), Human Ethical Committee (Ref. no. 101/HEC/2020). The patients/participants provided their written informed consent to participate in this study.

## Author Contributions

Conceptualization: SR. Methodology: KS, SD, AG, AJ, AA, SC, SS, GB, SSG, OS, and KA. Formal analysis: SR, SD, and AG. Resources: SR, JK, PP, RS, and AP. Data curation: AG and SD. Writing—original draft: KS, SD, and AG. Writing—review and editing: SR, KS, SD, and AG. Supervision: SR and AP. Project administration: SR and AP. Funding acquisition: AP. All authors contributed to the article and approved the submitted version.

## Funding

The work is supported by Science and Engineering Research Board, India (EMR/2016/000717), Department of Biotechnology, Ministry of Science and Technology (BT/PR15908/MED/12/725/2016) for providing the funding, and ILS intramural grant for supporting sequencing resources. 

## Conflict of Interest

The authors declare that the research was conducted in the absence of any commercial or financial relationships that could be construed as a potential conflict of interest.

## Publisher’s Note

All claims expressed in this article are solely those of the authors and do not necessarily represent those of their affiliated organizations, or those of the publisher, the editors and the reviewers. Any product that may be evaluated in this article, or claim that may be made by its manufacturer, is not guaranteed or endorsed by the publisher.
