# Supplementary figures and images for: Single-Cell Immunogenomic Approach Identified SARS-CoV-2 Protective Immune Signatures in Asymptomatic Direct Contacts of COVID-19 Cases
Source: Front Immunol. 2021 Nov 25;12:733539. doi: 10.3389/fimmu.2021.733539 (PMC8660575; doi:10.3389/fimmu.2021.733539)

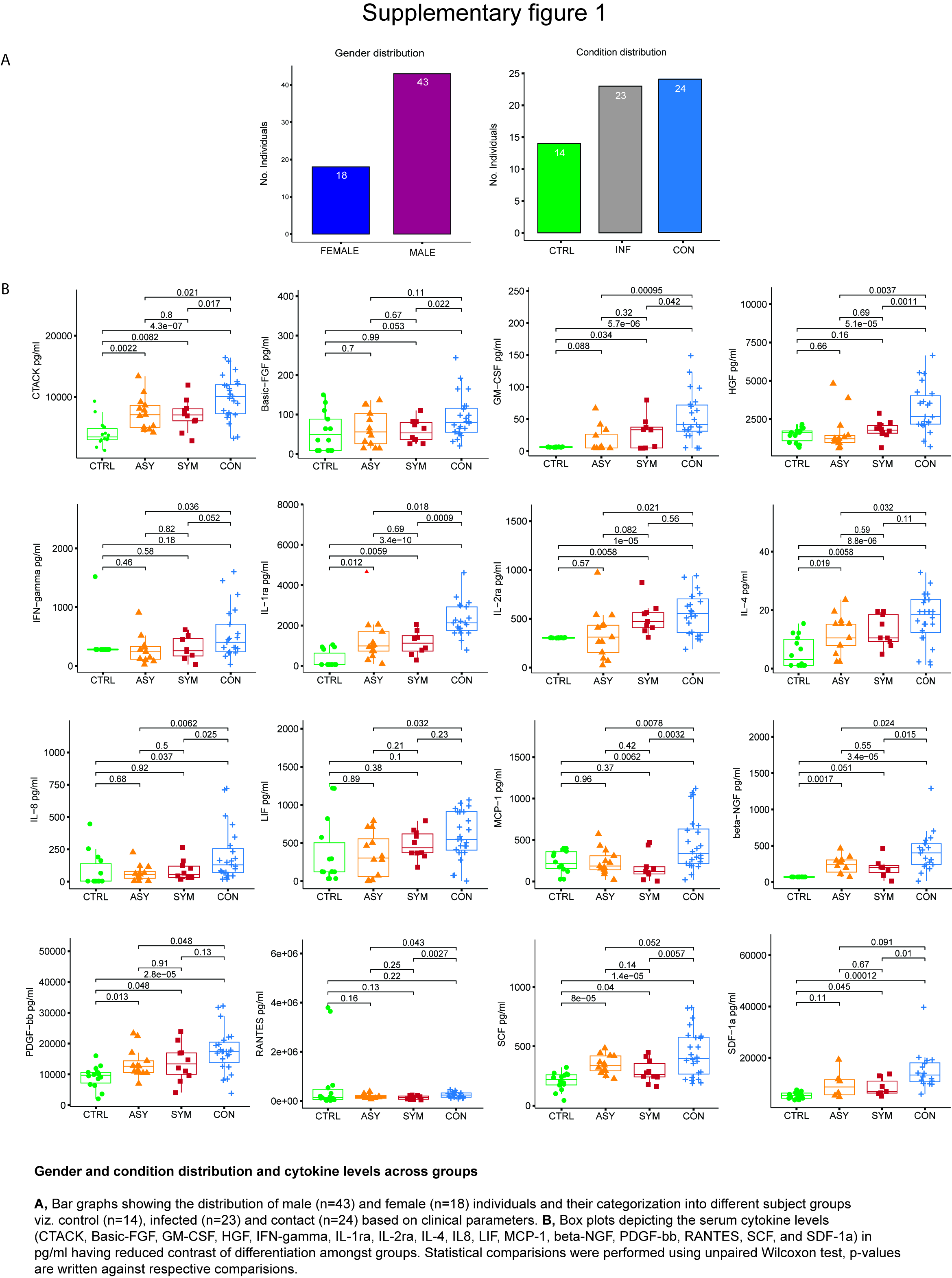

Supplement: Supplementary file 2 [file Image_1.tif]

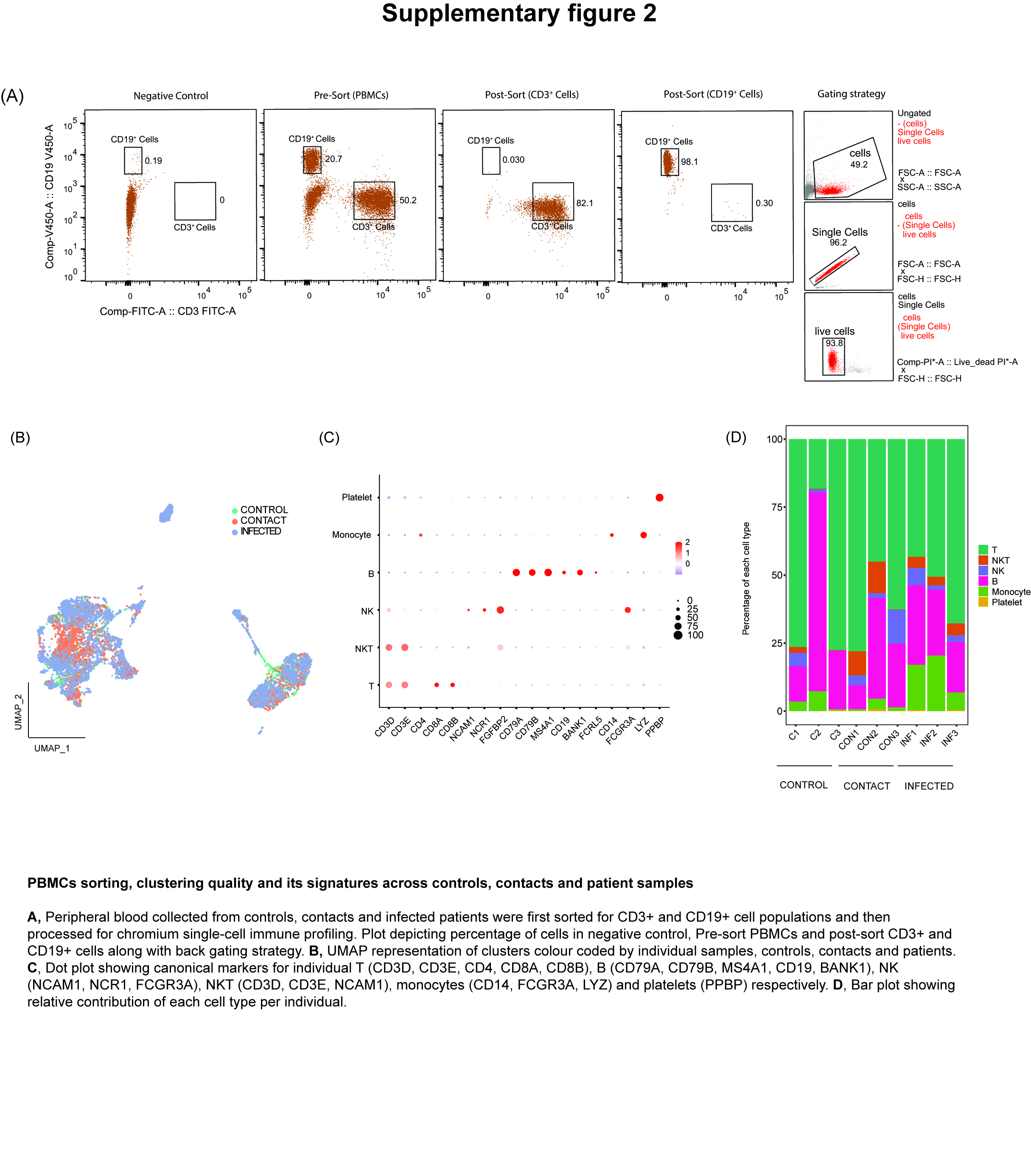

Supplement: Supplementary file 3 [file Image_2.tif]

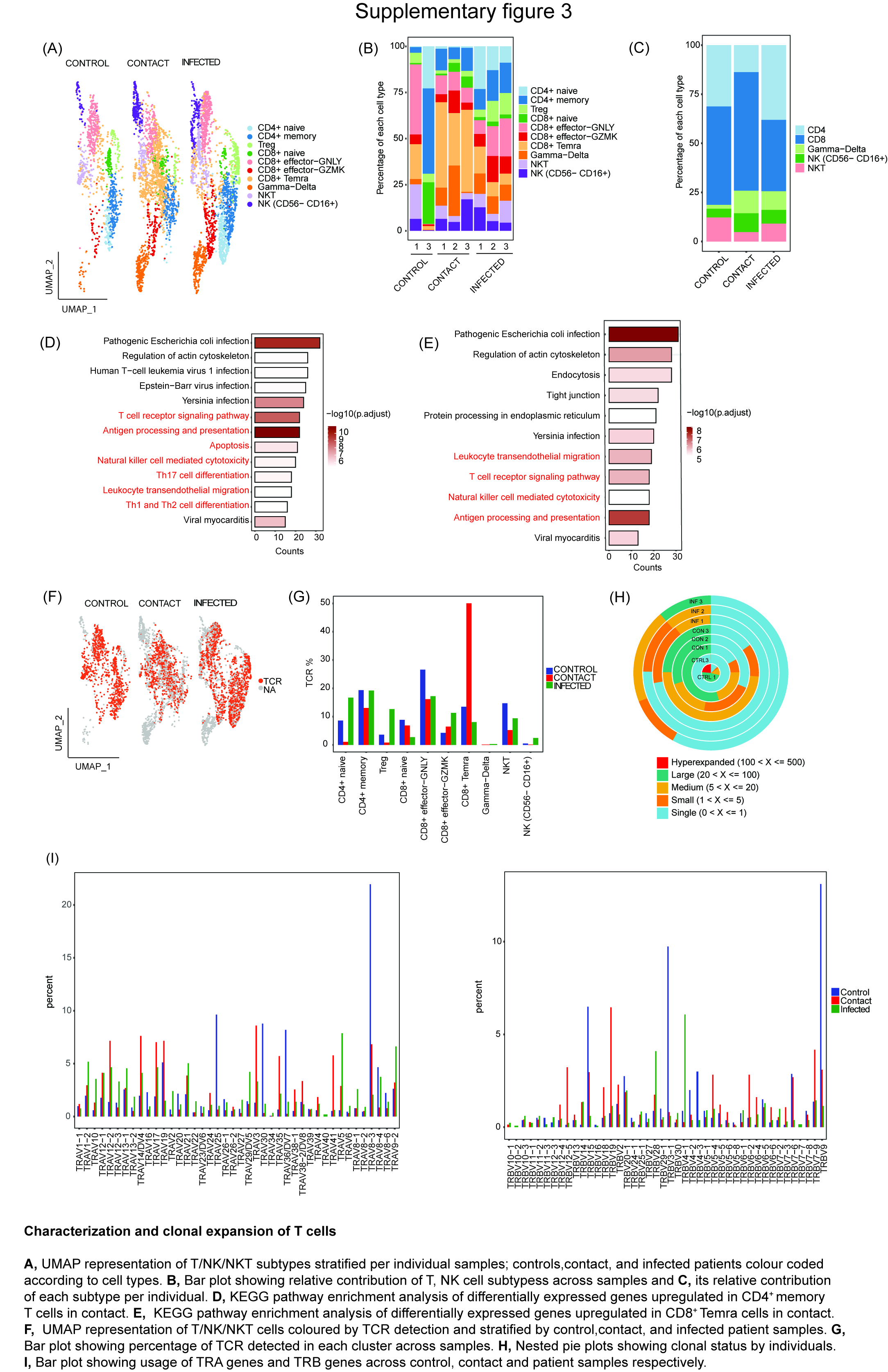

Supplement: Supplementary file 4 [file Image_3.tif]

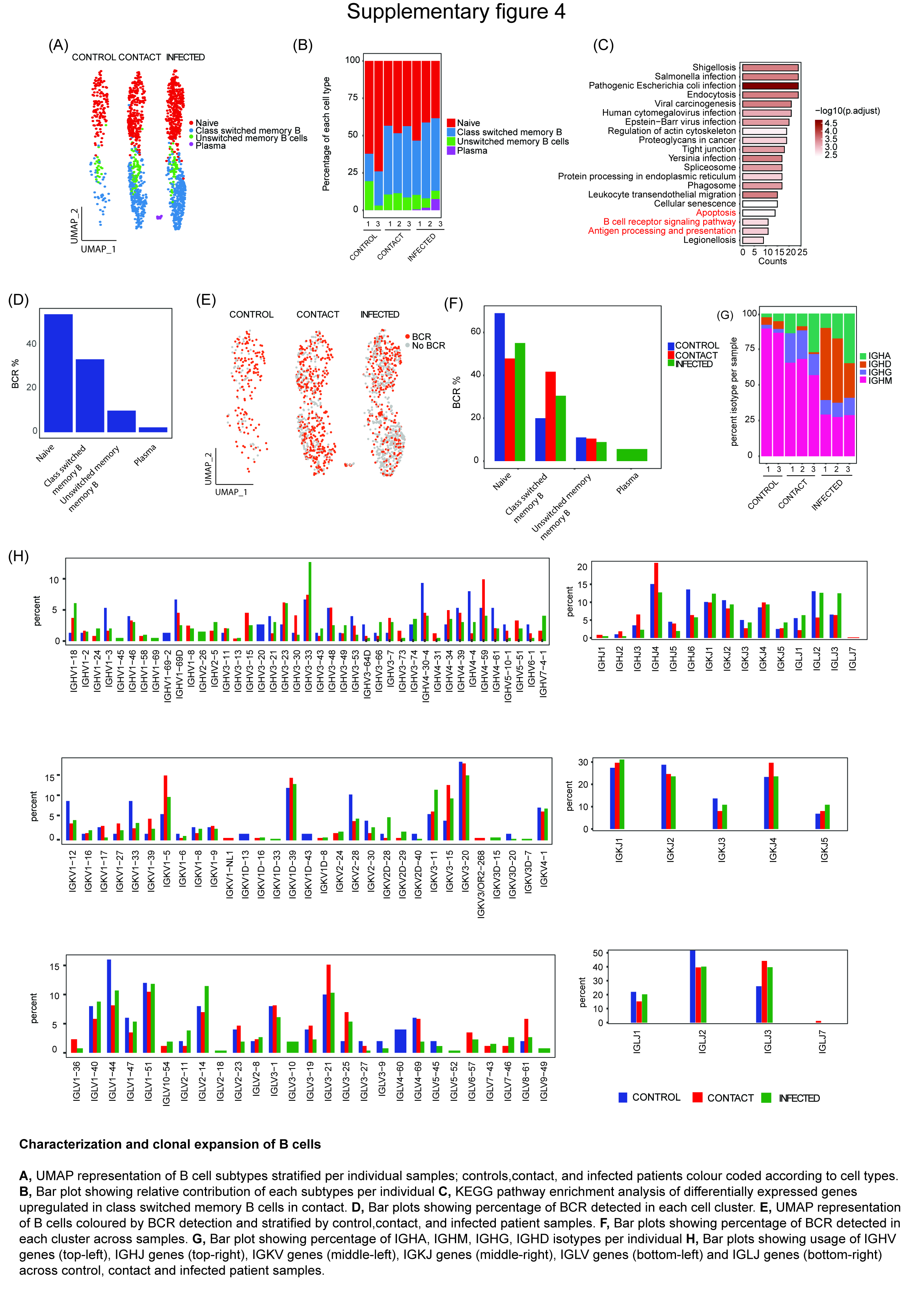

Supplement: Supplementary file 5 [file Image_4.tif]

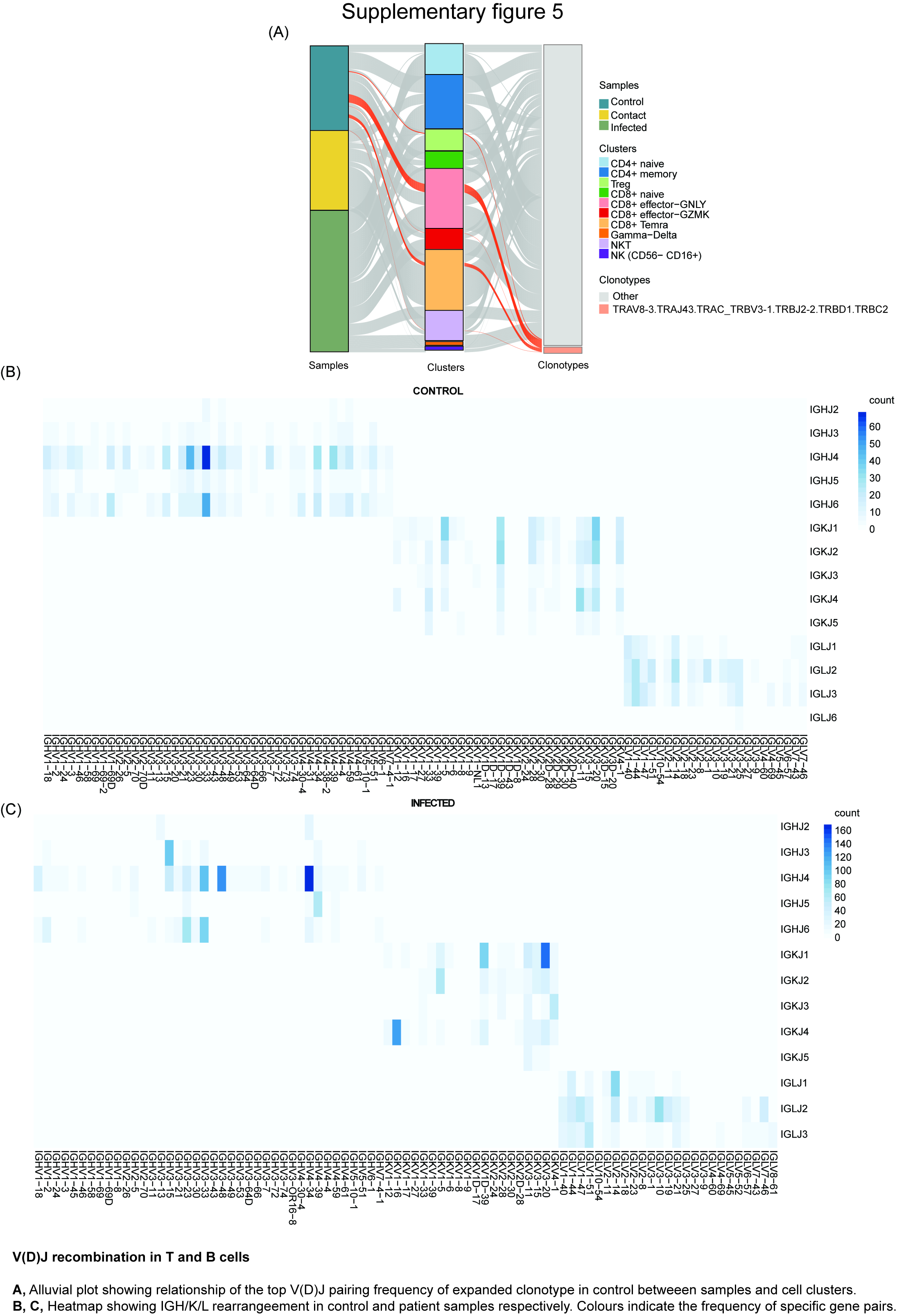

Supplement: Supplementary file 6 [file Image_5.tif]
